# Improving Surgical Triage Documentation Through Implementation of a Care of the Critically Ill Surgical Patient (CCrISP)-Based Proforma: A Quality Improvement Project

**DOI:** 10.7759/cureus.114965

**Published:** 2026-08-22

**Authors:** Sukhbir Sira, Michael Famewo, Yoosuf Y Ibrahim

**Affiliations:** 1 General Surgery, NHS Grampian, Aberdeen, GBR; 2 Renal Medicine, Aberdeen Royal Infirmary, Aberdeen, GBR

**Keywords:** ccrisp, clinical governance, patient safety culture, quality improvement projects, surgical admissions, surgical triage

## Abstract

Aims/background

Timely assessment and accurate documentation of acutely unwell surgical patients are important for safe clinical decision-making and effective multidisciplinary communication. This quality improvement project was conducted at a rural district general hospital in the north of Scotland, where acute surgical referrals are assessed by clinicians of varying grades and experience, and the surgical team provides cross-cover for related specialties. Variation in documentation prompted the development of a standardised Surgical Take Proforma based on the Royal College of Surgeons of England Care of the Critically Ill Surgical Patient (CCrISP) framework. This project evaluated whether introduction of the proforma was associated with improved completeness and consistency of surgical triage documentation.

Methods

A two-cycle, closed-loop audit was undertaken within the Department of General Surgery at NHS Grampian. Cycle 1 reviewed 30 consecutive acute surgical referrals documented using existing clerking methods between 15 and 30 December 2024. At the start of Cycle 2, the CCrISP-based Surgical Take Proforma was introduced, and staff education was provided through a Microsoft Teams meeting and face-to-face discussions. Cycle 2 reviewed a further 30 consecutive acute surgical referrals documented using the proforma between 1 and 30 January 2025. Documentation was assessed against five predefined criteria: A-E assessment, sepsis-related assessment or score, patient stability, blood gas or lactate documentation, and a definitive or daily management plan. Descriptive statistics and exploratory two-sided Fisher’s exact tests were used to compare the two cycles.

Results

Documentation of an A-E assessment increased from 0% in Cycle 1 to 80% in Cycle 2, while documentation of patient stability increased from 26.7% to 93.3%. Documentation of a definitive or daily management plan increased from 60% to 100%, and documentation of a sepsis-related assessment or score increased from 0% to 46.7%. Blood gas or lactate documentation increased from 73.3% to 86.7%. Exploratory Fisher’s exact testing demonstrated statistically significant differences for A-E assessment, sepsis-related assessment or score, patient stability, and definitive or daily management planning, with p<0.001 for each comparison. The difference in blood gas or lactate documentation was not statistically significant (p=0.333).

Conclusion

Introduction of a standardised CCrISP-based Surgical Take Proforma was associated with improved completeness of surgical triage documentation across the five audited criteria. The proforma was subsequently incorporated into a Patient Management Record template, demonstrating local institutional uptake. The project did not directly assess clinical assessment accuracy, clinical decision-making, communication, patient outcomes, educational effects, or documentation burden. Further evaluation is required to determine the sustainability of these improvements and their relationship to clinical and educational outcomes.

## Introduction

The preliminary assessment of acutely unwell surgical patients represents a key component of emergency surgical care. Accurate documentation is essential for patient safety, seamless continuity of care, communication between the multidisciplinary team and medico-legal accountability. District general hospitals frequently receive referrals from multiple sources, including primary care, emergency departments, advanced nurse practitioners, and inpatient specialties. This diversity can result in variation in assessment practices and documentation quality, particularly when patients are reviewed by clinicians with differing levels of experience. This is especially challenging for rural-remote localities where there is a generalist surgical take rather than referrals being received by sub-specialties.

The Royal College of Surgeons of England Care of the Critically Ill Surgical Patient (CCrISP) programme provides a structured framework for assessing all critically ill surgical patients through systematic Airway, Breathing, Circulation, Disability and Exposure (A-E) assessment and management planning [1]. Standardised documentation based on this framework may improve consistency, facilitate clinical decision-making, and reduce omissions during patient assessment [2].

CCrISP is a training programme for surgical doctors [1]. The course covers the theoretical basis and practical skills required to manage critically ill surgical patients. It is managed by the Royal College of Surgeons of England. Currently, it is a mandatory training requirement for early career surgical trainees within the UK. CCrISP was designed by Mr. Iain Anderson, Senior Lecturer in Surgery at Manchester University, for the Royal College of Surgeons of England [1]. The most recent iteration is the fifth edition, which is currently in use. The programme includes a structured algorithm for assessing and managing acutely unwell surgical patients through systematic A-E assessment, identification of physiological instability, investigation of underlying pathology, escalation of care, and formulation of a management plan.

Previous studies have demonstrated the benefits of structured assessment tools in improving surgical documentation and patient care. Pitcher et al. reported significant improvements in the consideration of key aspects of surgical management following implementation of a ward-round checklist, with further gains in documentation quality after introduction of a structured documentation form and staff education [2]. These findings suggest that checklist-based interventions may reduce variation in recorded clinical information and support more systematic patient assessment, providing a rationale for the development of our CCrISP-based Surgical Take Proforma.

The importance of structured clinical documentation is also reflected in the General Medical Council’s Good medical practice 2024 guidance. The GMC states that doctors must contribute to continuity of care, communicate effectively with colleagues, keep patients safe, and ensure that formal records are clear, accurate, contemporaneous, and legible. Structured documentation may therefore support professional expectations for clinical record keeping, although this project assessed documentation completeness rather than compliance with GMC standards directly [3].

This quality improvement project is reported in accordance with the Standards for Quality Improvement Reporting Excellence (SQUIRE 2.0) guidelines. SQUIRE 2.0 provides a framework for transparent reporting of quality-improvement initiatives, including their context, intervention, evaluation methods, and interpretation [4].

This quality improvement project aimed to evaluate the completeness and consistency of documentation for acute surgical referrals before and after implementation of a standardised CCrISP-based Surgical Take Proforma. Documentation was assessed against five predefined criteria: documentation of an A-E assessment, inclusion of a sepsis-related assessment or score, documentation of patient stability, documentation of a blood gas or lactate result, and documentation of a definitive or daily management plan. The project evaluated documentation quality only; any effects on the quality of clinical assessment, clinical decision-making, communication, education, or patient outcomes were considered potential implications requiring further evaluation.

## Materials and methods

Study design

**Figure 1:**
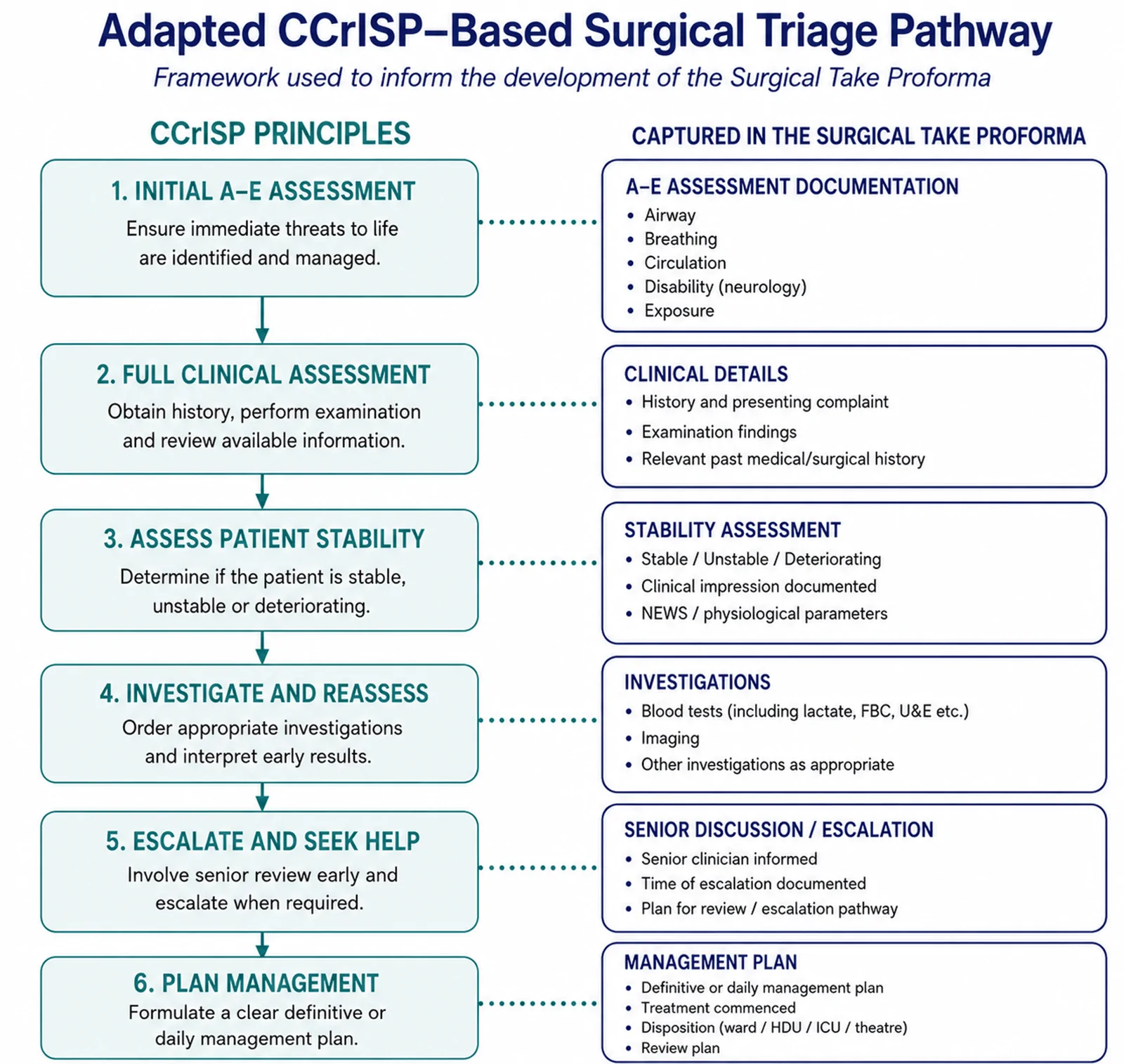
Adapted CCrISP-based surgical triage pathway CCrISP: Care of the Critically Ill Surgical Patient.

A closed-loop audit was conducted within the Department of General Surgery at NHS Grampian using the Model for Improvement framework. Audit standards were derived from the Royal College of Surgeons of England CCrISP algorithm (see Figure 1). Documentation was assessed against five predefined criteria derived from the locally adapted CCrISP-based proforma: documentation of an A-E assessment, documentation of a sepsis-related assessment or score, documentation of patient stability, documentation of a blood gas or lactate result, and documentation of a definitive or daily management plan. These criteria were selected because they represented core elements of the initial acute surgical assessment and were explicitly prompted within the proforma. The proforma itself also mandates inclusion of additional information relating to members of the team responsible for the patient, important clinical parameters relating to observations and biochemistry and additional relevant investigations. Figure 1 illustrates the key principles of the CCrISP framework and demonstrates how these informed the development of the Surgical Take Proforma. Core components include systematic A-E assessment, evaluation of physiological stability, investigation of underlying pathology, escalation of care, and formulation of a management plan. 

For the purposes of the audit, a criterion was considered present only when the relevant information was explicitly documented in the clinical record. An A-E assessment required documentation of assessment of the airway, breathing, circulation, disability, and exposure. Documentation of patient stability required an explicit statement regarding physiological stability or instability. A definitive or daily management plan required a documented plan addressing ongoing management, investigation, treatment, escalation, or disposition. Blood gas or lactate documentation required a recorded result. Documentation of sepsis assessment was considered present when the clinical record explicitly indicated that sepsis had been considered. This included a documented impression of sepsis, reference to Sepsis Six in the management plan, or documentation of a recognised sepsis-related score, such as qSOFA.

The project was conducted using principles from the Model for Improvement described by Langley et al. [5]. The initial audit cycle identified deficiencies in documentation of acute surgical triage against predefined CCrISP standards. A CCrISP-based Surgical Take Proforma was subsequently developed and introduced as the quality improvement intervention. The impact of this change was evaluated through a second audit cycle, consistent with a Plan-Do-Study-Act (PDSA) approach to iterative quality improvement [5]. 

Cycle 1 was conducted between 15 December 2024 and 30 December 2024 and included a retrospective review of 30 consecutive acute surgical referrals documented using existing clerking methods. Cases were identified sequentially rather than selected randomly. Referrals originated from general practitioners, the Emergency Department, hospital wards, community services, and medical wards. Elective admissions were excluded, and all included cases represented acute clinical presentations.

**Figure 2:**
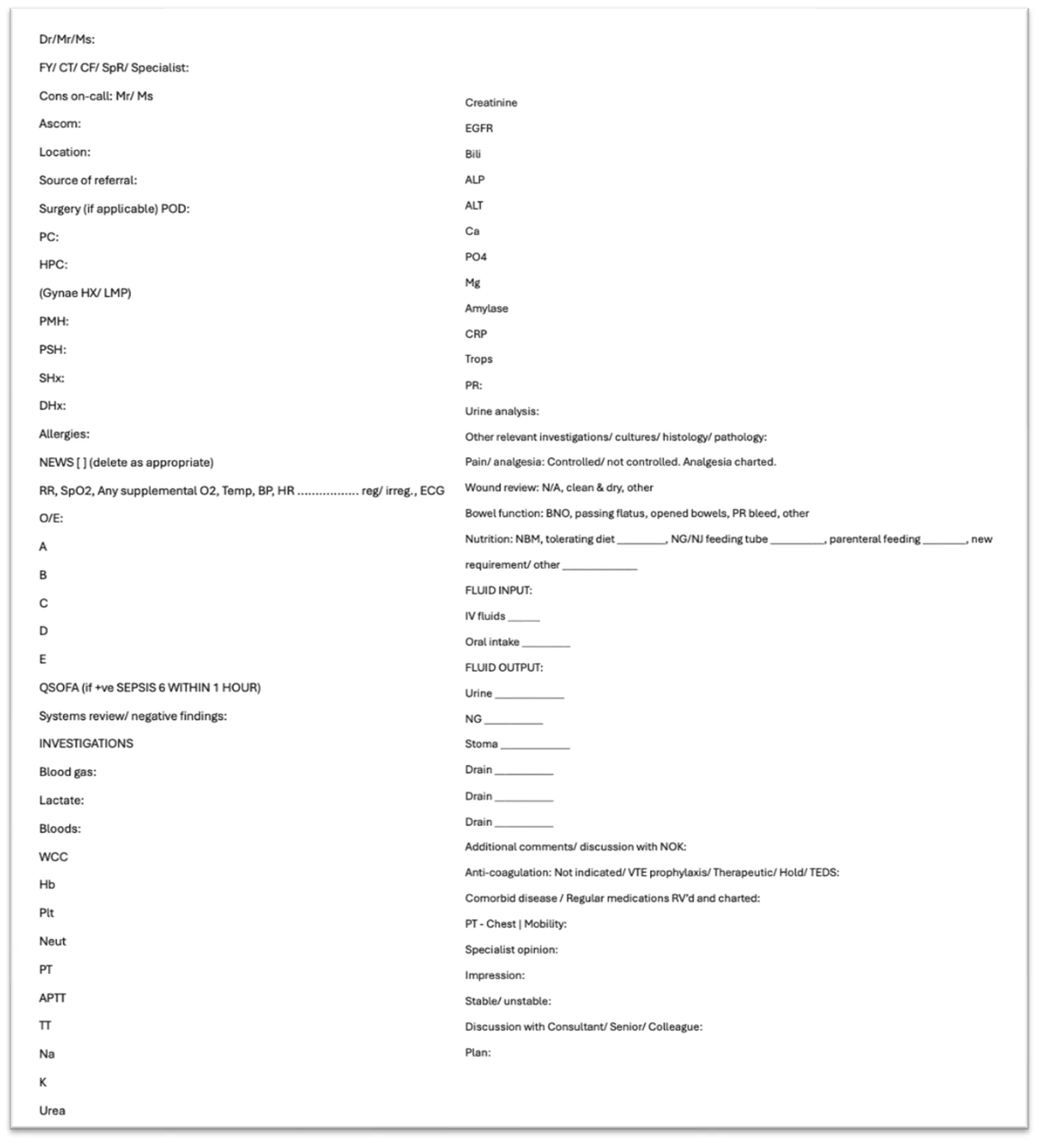
Standardized surgical take proforma introduced to improve the completeness and consistency of documentation during the initial assessment of acute surgical admissions

At the start of Cycle 2, the CCrISP-based Surgical Take Proforma (Figure 2) was introduced. Staff education was provided through a Microsoft Teams meeting and face-to-face discussions with surgical doctors accepting referrals. Cycle 2 was conducted between 1 January 2025 and 30 January 2025 and included a further 30 consecutive acute surgical referrals documented using the proforma. The proforma was completed by FY2 doctors, core surgical trainees, registrars, and surgical clinical fellows accepting referrals for the surgical service. As Cycle 2 commenced immediately after implementation, it represented the immediate post-implementation audit period, with no separate wash-in interval.

The proforma incorporated prompts for structured A-E assessment, sepsis assessment, assessment of patient stability, relevant investigations, and management planning. Findings from Cycle 1 were presented at the departmental multidisciplinary meeting and subsequently at a trust educational meeting. Following local review and appraisal, the intervention was incorporated into an electronic Patient Management Record (PMR) template.

Data analysis 

Data were collected using anonymised audit forms and entered into a Microsoft Excel spreadsheet (Microsoft Corporation, USA) for analysis. Compliance with each audit criterion was calculated as the percentage of reviewed records meeting that criterion. The analysis was based on 30 records in each audit cycle.

Each documentation criterion was coded as present or absent and compared between Cycle 1 and Cycle 2 using two-sided Fisher’s exact tests. Fisher’s exact testing was selected because of the small sample size and the presence of low or zero cell counts. Descriptive statistics were used to report the number and percentage of records meeting each criterion. Five documentation criteria were assessed; therefore, the statistical analyses were considered exploratory and the p-values were interpreted cautiously. Statistical significance was defined as a two-sided p-value of less than 0.05.

Ethical considerations and governance

This project was conducted as a closed-loop clinical audit and quality improvement initiative within the Department of Surgery of the hospital. The project was formally registered with the local Quality Improvement and Audit Team (QIAT) and undertaken in accordance with local clinical governance procedures.

As the project constituted service evaluation and quality improvement activity rather than research, formal Research Ethics Committee approval was not required under local NHS governance guidance. No patient identifiable information was collected during data collection or analysis. All data were anonymised and handled in accordance with NHS information governance and data protection policies.

The intervention involved the introduction of a documentation proforma designed to standardise assessment and record keeping and did not alter clinical management or expose patients to additional risk. The project was therefore considered a low-risk quality improvement initiative focused on enhancing documentation quality, communication, and patient safety. The clinical records were independently assessed by two members of the project team using anonymised audit data. A standardised extraction form was used during Cycle 2 but was not available during Cycle 1. Any disagreements were resolved through discussion and re-review of the relevant record. Formal inter-rater reliability was not assessed.

## Results

Improvements were observed across all five predefined documentation criteria following implementation of the Surgical Take Proforma. Documentation of a structured A-E assessment increased from 0% in Cycle 1 to 80% in Cycle 2. Documentation of patient stability increased from 26.7% to 93.3%, while documentation of a definitive or daily management plan increased from 60% to 100%. Documentation of a sepsis-related assessment or score increased from 0% to 46.7%. Documentation of a blood gas or lactate result increased from 73.3% to 86.7% (Table 1).

**Table 1:** Documentation quality before and after proforma implementation

Criterion	Cycle 1	Cycle 2	Fisher’s exact p-value
A–E assessment documented	0/30 (0%)	24/30 (80%)	<0.001
Sepsis-related assessment or score documented	0/30 (0%)	14/30 (46.7%)	<0.001
Patient stability documented	8/30 (26.7%)	28/30 (93.3%)	<0.001
Definitive/daily management plan documented	18/30 (60%)	30/30 (100%)	<0.001
Blood gas or lactate documented	22/30 (73.3%)	26/30 (86.7%)	0.333

Exploratory two-sided Fisher’s exact testing demonstrated statistically significant differences between Cycle 1 and Cycle 2 for documentation of an A-E assessment, a sepsis-related assessment or score, patient stability, and a definitive or daily management plan, with p<0.001 for each comparison. The increase in documentation of a blood gas or lactate result was not statistically significant (p=0.333). These analyses were exploratory and should be interpreted cautiously because of the small sample size, the use of an uncontrolled before-and-after design, and the absence of adjustment for multiple comparisons.

These findings represent changes in recorded documentation and do not directly establish improvements in the accuracy of clinical assessment, clinical decision-making, communication, education, or patient outcomes.

Although the audit formally assessed only five predefined documentation domains, qualitative review of the completed proformas suggested broader improvements in the overall organisation and comprehensiveness of clinical records. The proforma provided structured prompts for key aspects of the initial surgical assessment, including presenting complaint, relevant history, physiological assessment, investigation review, fluid balance, nutritional status, escalation planning, and multidisciplinary communication. Completion of these sections appeared to produce more systematic and standardised documentation compared with the free-text clerkings observed during the baseline audit. These observations were not prospectively defined outcome measures and were therefore not formally analysed; however, they suggest that the impact of the intervention may extend beyond the specific documentation parameters evaluated.

## Discussion

This project found that implementation of a CCrISP-based proforma was associated with improved completeness and consistency of surgical triage documentation. The largest increases were observed in documentation of an A-E assessment and patient stability, while documentation of a definitive or daily management plan reached 100% in Cycle 2. Because the project assessed documentation rather than clinical performance or patient outcomes, the findings demonstrate improvement in recorded information but do not establish that the underlying clinical assessments or decisions were more accurate or effective. These findings also suggest that structured documentation tools reduce variability in clinical practice and provide clinicians with prompts to perform comprehensive assessments [6,7]. The proforma increased the recording of the audited sepsis-related parameters. Given the observational before-and-after design, this finding should be interpreted as improved documentation rather than evidence of improved sepsis recognition or management. The proforma may also have had educational value by providing junior and non-specialist clinicians with a structured framework for documenting acute surgical assessments. However, educational outcomes were not directly measured in this project. Standardised tools can therefore function both as patient-safety interventions and educational resources [6]. 

The improvements observed following implementation of the proforma may be explained by its function as a “considerative checklist,” as described by Herring and Caldwell [6]. By embedding these prompts within routine documentation, the proforma may have functioned as a cognitive aid that supported a more consistent approach to surgical triage and reduced omissions in recorded information [6]. Treloar et al. similarly proposed that checklists function as aids that support decision-making and reduce omissions in patient care [7]. Thompson et al. demonstrated that the introduction of a post-take ward round proforma improved the completeness of clinical documentation and facilitated communication between healthcare professionals [8]. Patel et al. showed that a proforma can actively shape the assessment itself, behaving as a clinical prompt [9]. By embedding CCrISP principles within a standardised proforma, the intervention provided clinicians with a reproducible framework for assessment of acutely unwell surgical patients. The structured format may provide a useful framework for trainee learning, systematic patient evaluation, escalation planning, and formulation of management plans. These potential educational effects were not directly assessed and require further evaluation [9,10]. The educational benefits of the intervention may also be explained by findings from the systematic review by Khalaf and Khan, which identified lack of structure and competing service pressures as common barriers to learning [10].

A notable outcome of this project was the adoption of the proforma within the department, followed by the development of a PMR template based on the audit findings. This indicates local institutional uptake but does not establish long-term sustainability. Further evaluation is warranted to determine whether documentation improvements are maintained over time and whether they are associated with measurable changes in patient outcomes, multidisciplinary communication, and trainee development [8,11]. While the impact on clinical reasoning and decision-making was not formally evaluated, these areas represent potential additional benefits that warrant further exploration [6,7,10].

The authors noted that reviewing patient records following implementation of the proforma was easier because documentation was more uniform, clearer, and more succinct. Although this observation was not formally measured, it suggests that standardized documentation may facilitate case review, clinical handover, and future quality improvement activities [8,11].

An interesting study by Galloway et al. [11] evaluated documentation quality after a hospital transitioned from paper-based post-take ward round forms to electronic free-text records. The authors found substantial omissions in key clinical information, including escalation plans, frailty scores, social history, and predicted discharge dates. To address this, they introduced checklists, prompt cards, and ultimately an electronic proforma [11]. Their findings reinforce the present study by demonstrating that structured documentation remains valuable even within electronic health record systems, where reliance on free-text documentation alone may result in omission of important clinical information [11].

Limitations

This project has several limitations. It was a small, single-centre, uncontrolled before-and-after audit conducted in a rural district general hospital. The observed improvements cannot be attributed solely to the proforma, as staff education was delivered as part of the intervention. Secular changes, differences in clinicians or patient case mix, changes in workload, and a Hawthorne effect cannot therefore be excluded.

The project measured documentation rather than clinical performance or patient outcomes. Recording an A-E assessment does not establish that the assessment was complete or accurate, and improved documentation does not necessarily demonstrate improvements in patient safety, clinical decision-making, communication, or clinical outcomes. Each cycle included 30 records. Although exploratory Fisher’s exact tests were used to compare the two cycles, the study was not prospectively powered for hypothesis testing, confidence intervals were not reported, and no adjustment for multiple comparisons was applied. The statistical findings should therefore be interpreted cautiously and do not establish a causal effect of the proforma.

Patient-centred outcomes were not collected during this audit, which focused primarily on documentation completeness. Therefore, the project cannot determine whether the observed improvement in documentation was associated with changes in clinical outcomes, such as escalation to critical care, length of hospital stay, emergency intervention, complications, readmission, or mortality. Future prospective or retrospective evaluation should incorporate patient outcomes alongside documentation measures and account for differences in patient case mix, illness severity, and clinician factors. This would help establish whether improved documentation translates into meaningful changes in patient care and safety.

A further limitation is that the record reviewers were members of the project team and were aware of the intervention. Although both reviewers independently assessed every record and disagreements were resolved through discussion and re-review, a standardised extraction form was used only during Cycle 2 and not during Cycle 1. Formal inter-rater reliability was not assessed, which may have introduced measurement variability. The absence of blinded assessment may have introduced reviewer bias. In addition, the two cohorts may have differed in clinician experience, referral source, workload, or case mix. Although consecutive referrals were reviewed, randomisation was not used, and comparability between the two cycles cannot be guaranteed.

Longer-term compliance and sustainability were not assessed after the initial implementation period. Although subsequent incorporation of the intervention into an electronic PMR suggests institutional uptake, this does not establish sustained adherence over time.

Finally, balancing measures were not collected. The project did not assess completion time, documentation burden, clinician acceptability, or whether the template resulted in repetitive or “tick-box” documentation. Patient outcomes, escalation of care, communication, and educational effects were also not directly assessed.

## Conclusions

The introduction of a standardised CCrISP-based Surgical Take Proforma was associated with improved completeness of surgical triage documentation across the five audited criteria. The proforma was subsequently incorporated into a PMR template, demonstrating local institutional uptake. However, this project did not directly assess clinical assessment accuracy, clinical decision-making, multidisciplinary communication, patient outcomes, educational effects, or documentation burden.

Future work should evaluate whether improvements in documentation are sustained over time and whether they are associated with measurable changes in clinical outcomes, including earlier recognition of deterioration, timelier escalation of care, and reduced delays in management. The educational impact of the template could also be explored through trainee feedback and assessment of confidence in managing acutely unwell surgical patients. Regular re-audit following staff rotation and departmental changes may help assess continued compliance with CCrISP principles and identify opportunities for further refinement. Adaptation of the template for relevant subspecialty services, including trauma and orthopaedics, could also be explored to assess its wider applicability across acute surgical services.
